# Continuous Sedation in Palliative Care in Portugal: A Prospective Multicentric Study

**DOI:** 10.1177/08258597241256874

**Published:** 2024-05-25

**Authors:** José António Ferraz-Gonçalves, Alice Flores, Ana Abreu Silva, Ana Simões, Carmen Pais, Clarisse Melo, Diana Pirra, Dora Coelho, Lília Conde, Lorena Real, Madalena Feio, Manuel Barbosa, Maria de Lurdes Martins, Marlene Areias, Rafael Muñoz-Romero, Rita Cunha Ferreira, Susete Freitas

**Affiliations:** 1 Faculty of Medicine, Porto University, Porto, Portugal; 2 Department of Palliative Care, 466919 Unidade Local de Saúde do Nordeste, Macedo de Cavaleiros, Portugal; 3 Department of Palliative Care, Serviço de Saúde da Região Autónoma da Madeira (SESARAM), Funchal, Portugal; 4 Hospital Palliative Care Team and Home Care Unit, 37838 Portuguese Oncology Institute of Lisbon, Lisbon, Portugal; 5 Clinical Academic Center of Trás-os-Montes and Alto Douro – Professor Doctor Nuno Grande – CACTMAD, Vila Real, Portugal; 6 Community Team of Palliative Care, ACES Lisboa Ocidental e Oeiras, Lisbon, Portugal; 7 Department of Palliative Care, 467109 Hospital Santa Luzia, Elvas, Portugal; 8 Department of Palliative Care, Centro Hospitalar Universitário do Algarve, Faro, Portugal; 9 Community Team of Palliative Care, Maia/Valongo, Portugal

**Keywords:** palliative sedation, continuous sedation, refractory symptoms, delirium, midazolam

## Abstract

**Objective:** This study aimed to survey the practice of palliative sedation in Portugal, where data on this subject were lacking. **Methods:** This was a prospective multicentric study that included all patients admitted to each team that agreed to participate. Patients were followed until death, discharge, or after 3 months of follow-up. **Results:** The study included 8 teams: 4 as palliative care units (PCU), 1 as a hospital palliative care team (HPCT), 2 as home care (HC), and 1 as HPCT and HC. Of the 361 patients enrolled, 52% were male, the median age was 76 years, and 285 (79%) had cancer. Continuous sedation was undergone by 49 (14%) patients: 26 (53%) were male, and the median age was 76. Most patients, 46 (94%), had an oncological diagnosis. Only in a minority of cases, the family, 16 (33%), or the patient, 5 (10%), participated in the decision to sedate. Delirium was the most frequent symptom leading to sedation. The medication most used was midazolam (65%). In the multivariable analysis, only age and the combined score were independently associated with sedation; patients <76 years and those with higher levels of suffering had a higher probability of being sedated. **Conclusions:** The practice of continuous palliative sedation in Portugal is within the range reported in other studies. One particularly relevant point was the low participation of patients and their families in the decision-making process. Each team must have a deep discussion on this aspect.

## Introduction

Palliative care (PC) aims to allow patients with advanced and progressive chronic diseases to live the best and most actively possible.^1^ Nevertheless, there are situations where it is not possible to control symptoms without interfering with the consciousness level. Those symptoms “cannot be adequately controlled despite aggressive efforts to identify a tolerable therapy that does not compromise consciousness.”^2^ According to Cherny et al, those symptoms are named “refractory” and must be distinguished from “the difficult symptom” that could potentially respond within a tolerable time frame to noninvasive or invasive interventions and yield adequate relief and preserved consciousness without excessive adverse effects.^2^ That distinction may be difficult, mainly concerning psychological or existential problems.^3,4^ There is no red line between refractory and difficult symptoms. The distinction depends on several factors, including the professionals’ competence, care setting, and available resources. All those factors vary across PC services.

About 30 years ago, Ventaffrida et al drew attention to the issue of sedation at the end of life when they reported that 52.5% of the patients of their service were sedated.^5^ That study led researchers to perform others on that topic, which showed percentages of sedated patients from 1.3% to 52.5%.^6^ In a study carried out in Portugal by Ferraz-Gonçalves et al^7^ about the last 48 hours of life of patients in a PC unit, 10% of patients were sedated deeply according to the Chater et al definition.^8^ In another Portuguese study from 2011, with the participation of 4 of the 19 teams identified at that time through the Portuguese Association for PC site, only 8% were continuously sedated until death.^9^ However, the different percentages of sedated patients through the studies do not necessarily reflect significant differences in the practice because the meaning of sedation was not clearly defined in many studies; some studies do not even define it.^10^

After the previous study on sedation performed in Portugal mentioned above, PC evolved with more teams and more experienced PC teams. This study intends to check if and how Portuguese teams are presently dealing with this critical aspect of care.

## Methods

### Study Design

This study was prospective, observational, and multicentric. One doctor and one nurse conducted the study in each team, although other members of the teams participated in data collection.

### Setting and Participants

All patients admitted to any care setting (PC unit, hospital PC team, or home care [HC]) and with any diagnosis (cancer or non-cancer) for 3 consecutive months were enrolled in the study from March to December 2022. The patients were followed until death, discharge, or when a period of follow-up of 3 months was completed.

### Data Collection

A doctor of the PC team assessed all patients at admission according to the study protocol. During the follow-up, the team nurses completed the consciousness and comfort/discomfort assessment.

At admission, the demographic data of all patients were collected. Other data included the primary diagnosis and the Charlson comorbidity index.^11^ The setting of care, the total burden of diseases, including primary disease, the motive for sedating, who decided on sedation, who gave the consent for sedation, when sedation was started, feeding and hydration during sedation, and finished, and if death was deemed peaceful were also recorded. The Palliative performance scale was used to assess the performance status.^12^ Delirium was screened with a validated Portuguese version of the Confusion Assessment Method.^13^

The symptom assessment of patients with and without cognitive failure was different. For patients without cognitive impairment, the authors used the Initial Assessment of Patients Without Cognitive Failure Admitted in PC Scale (IAPAPCS).^14,15^ No tools for patients with cognitive failure were found explicitly developed for PC to assess comfort/discomfort. Therefore, sedation studies use tools developed for dementias, such as the DS-DAT.^16^ Hence, a scale was developed and submitted to facial validation by 8 doctors and nurses working in PC who did not participate in this study. The scale was constructed with four levels (assessment after 5 minutes of observation): 1, no signs of discomfort; 2, rare signs of discomfort; 3, frequent signs of discomfort; 4, constant or almost constant signs of discomfort. This scale was used in the initial assessment and during the sedation period with intervals of 12 hours, except at home, where the HC team assessed patients once a day.

A combined score was built to estimate the level of suffering in patients whose symptoms could be assessed directly and those where that was not possible due to cognitive failure. The sum of the IAPAPCS scores was divided by the quartiles, and each quartile was deemed equivalent to each level of the discomfort scale described above.

The consciousness level was initially assessed during the sedation period with the Consciousness Scale for PC (CSPC),^17^ which has received one of the highest ratings on psychometric properties according to a recent systematic review evaluating instruments to monitor the level of consciousness on palliative patients.^18^ This scale has six consciousness levels, which are: 1, awake; when called by their name: 2, awakens and stays awake during the conversation; 3, awakens but falls asleep during the conversation, 4, reacts with movement or brief eye-opening, but without eye contact; trapezius muscle pinching: 5, reacts; 6, does not react.

The Anatomical Therapeutic Chemical Classification of the World Health Organization was used for categorizing medication: Opioids (N02A) and psycholeptics (N05). The ophthalmic, cutaneous, and rescue medications were excluded from computing the total number of medications.

In this study, the objective was to study continuous sedation. Therefore, episodes of intermittent or emergent sedation and sedation resulting from the adverse effects of medication also were not included. The definition of sedation used was the same as in the previous study in Portugal: the intentional administration of sedative medications for controlling refractory symptoms, except insomnia, independently of the consciousness level reached. The level of consciousness reached was not essential, as the objective was suffering control independently of the level.

### Statistical Analysis

Regarding statistics, initially, an exploratory analysis of the data was performed to describe the sample. The Chi-squared test was used to compare variables in different settings. A logistic regression was used for univariable and multivariable analyses to assess the independent factors associated with continuous sedation at admission. The level of significance was deemed to be 0.05. The 29.0 version of the IBM SPSS statistical software was used to analyze the data.

### Ethical Considerations

The study was approved by the ethics committee at each study site.

Consent was obtained from the patient or the family to utilize the patient's data for the study according to the patient's cognitive competence.

## Results

The study was performed between March and December 2022. The study included 8 teams: 4 as PC units (PCU), 1 as hospital PC team (HPCT), 2 as HC, and 1 as HPCT and HC. Patients enrolled were 361, 230 (64%) in PCU, 75 (21%) in HPCT, and 56 (15%) in HC. Most patients, 52%, were male, and the median age was 76 years (18 to 102). In 285 (79%) patients, the primary diagnosis was oncological, and in 76 (21%) non-oncological. Table 1 shows the demographic data.

**Table 1.** Demographic Data at Admission in Sedated and Non-Sedated Patients.

	Sedated (49) n (%)	Non-sedated (312) n (%)	Total (361) n (%)
Gender			
Male	26 (53)	163 (52)	189 (52)
Female	23 (47)	149 (48)	172 (48)
Age			
<65	15 (31)	68 (22)	83 (23)
65-75	18 (37)	70 (24)	88 (24)
76-84	10 (20)	86 (28)	96 (27)
≥85	6 (12)	88 (28)	94 (26)
Main disease			
Oncol	46 (94)	239 (77)	285 (79)
Non-oncol	3 (6)	73 (24)	76 (21)
Type of team			
PCU	32 (66))	198 (64)	230 (63)
HPCT	12 (25)	63 (20)	75 (21)
HC	5 (10)	51 (16)	56 (16)
Symptom assessment possible			
Yes	23 (47)	173 (55)	196 (54)
No	26 (53)	139 (45)	165 (46)
Comorbidities (including primary disease)			
≤2	8 (16)	82 (26)	90 (25)
3-6	7 (14)	64 (21)	71 (20)
7-8	13 (27)	71 (23)	84 (23)
≥9	21 (43)	95 (30)	116 (32)
Consciousness level			
1	34 (69)	213 (68)	247 (68)
2	2 (4)	40 (13)	42 (12)
3	10 (20)	26 (8)	36 (10)
≥4	3 (6)	33 (11)	36 (10)
Confusion assessment method			
Delirium	17 (35)	67 (22)	84 (23)
No delirium	27 (55)	216 (69)	243 (67)
Unassessed	5 (10)	29 (9)	34 (9)
Palliative performance scale			
≤30	38 (78)	213 (68)	251 (70)
>30	11 (22)	99 (32)	110 (31)
Combined score			
1	7 (14)	89 (29)	96 (27)
2	7 (14)	109 (35)	116 (32)
3	30 (61)	81 (26)	111 (31)
4	5 (10)	33 (11)	38 (11)
Opioids			
Yes	36 (74)	191 (61)	227 (63)
No	13 (27)	121 (39)	134 (37)
Psycholeptics			
Yes	28 (57)	168 (54)	196 (54)
No	21 (43)	114 (46)	165 (46)
Oxygen			
Yes	14 (29)	79 (25)	93 (26)
No	35 (71)	233 (75)	268 (74)
Number of medications			
≤5	11 (22)	80 (26)	91 (25)
6-7	11 (24)	76 (24)	87 (24)
8-9	13 (27)	70 (22)	83 (23)
≥10	14 (29)	86 (28)	100 (28)

Forty-nine (14%) patients underwent continuous sedation: 32 (65%) in PCU, 12 (24%) in HPCT, and 5 (10%) in HC. Twenty-six (53%) were male, and the median age was 76. Most patients, 46 (94%), had an oncological diagnosis. No statistically significant differences were found among the teams concerning sex (*P* = .732), age (*P* = .113), and continuous sedation (*P* = .489). However, significant differences were found in the type of primary disease (*P* < .001) and the possibility of assessing symptoms (*P* < .001) (Table 2). All the sedated patients were deemed as having refractory symptoms in 47 (96%) cases by the team and by the doctor alone in 2 (4%) cases; in some cases, other professionals were also involved: psychologist/psychiatrist in 6 (12%) cases, an external medical consultant in 2 (4%) cases and in 1 (2%) spiritual assistant.

**Table 2.** Variables in the Different PC Settings.

	PCU n (%)	HPCT n (%)	HC n (%)	Total	*P*
Sex					
Male	124 (54)	37 (49)	28 (50)	189 (52)	.732
Female	106 (46)	38 (51)	28 (50)	172 (48)	
Age					
<65	55 (24)	22 (29)	6 (11)	83 (23)	.113
65-75	53 (23)	20 (27)	15 (27)	88 (24)	
76-84	67 (29)	14 (19)	15 (27)	96 (27)	
≥85	55 (24)	19 (25)	20 (36)	94 (26)	
Main disease					
Oncol	169 (74)	70 (93)	46 (82)	285 (79)	**<** **.** **001**
Non-oncol	61 (27)	5 (7)	10 (18)	76 (21)	
Symptom assessment possible					
Yes	99 (43)	58 (77)	39 (70)	196 (54)	**<**.**001**
No	131 (57)	17 (23)	17 (30)	165 (46)	
Sedation (continuous)					
Yes	32 (14)	12 (16)	5 (9)	49 (14)	.489
No	198 (86)	63 (84)	51 (91)	312 (87)	

Abbreviations: HC, home care; HPCT, hospital PC team; PCU, PC unit hospital PC team.

Bold – significant variables.

The team decided to sedate in 40 (82%) cases and the doctor in 9 (18%) cases. Only in a minority of cases did the family, 16 (33%), or the patient, 5 (10%), participate in the decision. The symptom that more frequently led to sedation was delirium in 31 (63%) cases, followed by dyspnea and pain, but in several cases, a combination of problems motivated sedation. In only one case, the cause of sedation was psychological and existential suffering; in no other case, those problems were the isolated explanation, although they compounded the set of problems in several cases (Table 3).

**Table 3.** Causes of Sedation per Symptom.

Delirium	31	63%
Dyspnea	13	27%
Pain	11	22%
Psychological suffering	6	12%
Seizures	4	8%
Existential suffering	4	8%
Pruritus	1	2%
Nausea/vomiting	1	2%
Myoclonus	1	2%
Hemorrhage	1	2%

Totals are >100% because patients could experience more than 1 symptom contributing to the decision to sedate.

In univariable analysis, possible determinants of sedation at admission were age, the primary disease (oncological vs non-oncological), consciousness level, cognitive function, and level of suffering (Table 4). The gender, care setting, the Charlson comorbidity index, the possibility of assessing symptoms directly, the performance status, the use of opioids, psycholeptics, or oxygen, and the number of medications at admission were not determinants of sedation. In the multivariable analysis, only age and the combined score: the younger and those with higher suffering levels are more likely to be sedated (Table 5).

**Table 4.** Factors at Admission in Sedated and Non-Sedated Patients, Univariable Analysis.

Factor	OR	95.0% CI	*P*
Gender			
Male	1		
Female	0.97	0.53-1.77	.915
Age (quartiles)			
≥85	1		
76-84	1.71	0.59-4.90	.321
65-75	**3** **.** **77**	**1.42-10.01**	.**008**
<65	**3**.**24**	**1.19-8.78**	.**021**
Main disease			
Oncological	1		
Non-oncological	**0**.**21**	**0.07-0.71**	.**011**
Team type			
PCU	1		
HPCT	1.94	0.64-5.88	.239
HC	1.65	0.61-4.44	.323
Charlson comorbidity index (including the primary disease)				
≤2	1			
3-6	1.12	0.39-3.26	.834
7-8	1.88	0.74-4.79	.188
≥9	2.27	0.95-5.39	.064
Symptom assessment possible			
No	1		
Yes	0.71	0.39-1.30	.27
Consciousness level			
1	1		
2	0.31	0.07-1.36	.121
3	**2**.**41**	**1.07-5.44**	.**034**
≥4	0.57	0.17-1.96	.372
Confusion assessment method			
Delirium	1		
No delirium	**0**.**49**	**0.25-0.96**	.**037**
Not assessable	0.68	0.23-2.02	.486
Palliative performance scale			
≤30	1		
>30	0.62	0.31-1.27	.193
Combined score			
1	1		
2	0.82	0.28-2.42	.714
3	**4**.**71**	**1.96-11.31**	**<**.**001**
4	1.93	0.57-6.49	.290
Opioids			
No	1		
Yes	0.57	0.29-1.12	.102
Psycholeptics			
No	1		
Yes	0.88	0.48-1.61	.667
Number of medications			
≤5	1		
6-7	1.05	0.43-2.57	.910
8-9	1.35	0.57-3.21	.496
≥10	1.18	0.51-2.76	.696
Oxygen			
No	1		
Yes	1.18	0.60-2.31	.63

Abbreviations: CI, confidence interval; HC, home care; HPCT, hospital PC team; PCU, PC unit hospital PC team; OR, odds ratio.

Bold – significant variables.

**Table 5.** Factors at Admission in Sedated and Non-Sedated Patients, Multivariable Analysis.

	OR	95.0% CI		*P*
Age				
≥85	1			
76-84	1.58	0.53-4.68		.408
65-75	**4** **.** **73**	**1.70-13.15**		.**003**
<65	**4**.**01**	**1.39-11.52**		.**010**
Combined score				
1	1			
2	0.86	0.29-2.57		.785
3	**5**.**76**	**2.33-14.24**		**<**.**001**
4	1.69	0.49-5.81		.407
				

Bold – significant variables.

Abbreviations: CI, confidence interval; OR, odds ratio.

Of the 49 patients sedated continuously, 48 died during sedation. The patient who did not die began intending to be sedated until death, but the patient stabilized and could be discharged. The duration of sedation ranged from 5.83 to 807.50 hours, with a median of 62.17 hours and interquartile (IQR) of 121.71 hours. During the sedation period, many patients could maintain oral feeding and hydration, usually in small portions and four even in normal portions. Artificial hydration was used in 20% of the patients, and enteric artificial feeding was used in only one patient. The median of the last measured consciousness level was 5 (range: 1-6; IQR:2), and the median comfort level was 1 (range: 1-4; IQR: 1). The most used medication was midazolam (88% of patients), alone (65%) or in combination with other medications (Table 6). The median daily dose of midazolam was 15 mg (range: 2.5-50 mg). Of the 48 patients who died while sedated, the death was deemed peaceful in 47 (98%).

**Table 6.** Medications Used for Sedation.

	N (%)
Midazolam	32 (65)
Levomepromazine	3 (6)
Haloperidol	2 (4)
Chlorpromazine	1 (2)
Midazolam + levomepromazine	4 (8)
Midazolam + haloperidol	4 (8)
Midazolam-quetiapine	1 (2)
Midazolam + levomepromazinel + phenobarbital	1 (2)
Midazolam + lorazepam + olanzapine	1 (2)
Total	49 (100)

## Discussion

The reported prevalence of palliative sedation varies widely; in one review ranged from 1.3% to 52.5%,^6 ^and in a more recent ranged from 0% to 55%.^19^ Some studies combine various types of sedation,^20,21^ and the percentage of continuous sedation can be challenging to see. In this study, the percentage of patients continuously sedated was 14%, which is in the lower part of the range. In the previous study in Portugal, only 6% were continuously sedated. However, the meaning of this difference needs to be clarified.

There were no significant differences among PCU, HPCT, and HC. Most studies concentrated on inpatients in PCUs, but palliative sedation is also feasible at home.^19^ Data about hospital PC teams is scarce. It was not possible to find recent studies focusing on this setting. Nevertheless, a few data show that it is not fundamentally different from a PCU.^22,23^ This finding is not unexpected, as patients are in a hospital followed by PC teams.

Most decisions were made after discussion among the care team members. The patient participated in the decision in only 10% and the family in 33%. These percentages are low, like those found in the other study on sedation performed in Portugal.^9^ Most guidelines mention informed consent as a requirement for continuous sedation, and some of them also include the family.^24^ When cognitively competent, patients should be involved in decision-making; their involvement differentiates between an ethical and an unethical process. Family can also be involved with the patient's agreement and when the patient cannot participate or at least be informed. This aspect should be further explored and discussed. Teams may use published guidelines like Ostgathe et al.^25^ However, although those guidelines can be a basis for reflection, teams should discuss this issue and develop guidelines that can be accepted by their members and the populations they serve.

Delirium, dyspnea, and pain were the more frequent reasons to sedate. In a recently published systematic review, those were also the more frequent causes.^24^ However, combining physical symptoms with psychological and existential suffering was common. Psychological and existential suffering are reasons particularly controversial for sedation.^3,4,26^ There are wide variations in the frequency of those sedation causes, and some reports do not mention them.^27^ This study reported a combination of psychological and existential suffering in only one case. In the other cases, those problems were combined with physical symptoms, which should not be unexpected as it is in keeping with the Cicely Saunders concept of total pain.^28^

Midazolam was used in most cases as it usually occurs and is often recommended, such as by the European Association for PC.^21,29,30^ In the previous Portuguese study, midazolam was also primarily used.^9^ Several other medications have been used for sedation, some as a second line when midazolam is not enough, such as levomepromazine, sometimes in combination, as in this study, and as a third step, sometimes phenobarbital or even propofol.^30^ In the present study, of those third-step medications, only phenobarbital was used in one patient.

Several studies concluded that sedation does not influence survival.^10,31^ Although studies did not show evidence that hydration and nutrition do not prolong life,^32^ prolonged deep sedation may precipitate death if not supported with hydration or even feeding. In this study, a few patients maintained regular food and liquid intake, reflecting the light level of sedation in those patients. However, most who could maintain oral feeding and drinking did it only in small portions. Artificial tube feeding was used in only one patient. The decision to maintain or continue hydration and nutrition should be independent of the decision to sedate. If a patient has stopped ingesting food and drinks, starting them after sedation does not make sense. If the patient maintained the ingestion, the decision should be individualized.

To the best of our knowledge, only one study addressed the patient-related determinants at the admission of the administration of continuous palliative sedation.^28^ In the present study, the independent factors resulting from the multivariable analysis were age and the level of suffering. In contrast, in that study, only the use of opioids at admission was significant. The hypothesis raised by the authors of that study was that the determinants could be the symptoms (pain or dyspnea) that justify the use of opioids and not the opioids themselves. In the present study, level 3 of the combined score reflecting a high level of suffering was significantly associated with sedation; level 4 was not, possibly because the number of patients in that level was much lower.

This study has some weaknesses. Although the number of centers that participated in this study was higher than in the previous one, the number is still low and may not represent the Portuguese reality. Nevertheless, this is the only recent approach to continuous sedation in Portugal. Researchers were not blind to the patient's situation; therefore, the absence of some bias is not guaranteed. The scale developed for assessing comfort/discomfort underwent only facial validation, which is insufficient to evaluate its validity and reliability. However, the lack of validated tools for PC can justify its use. Also, the combined score used in this study was not validated, but it seems useful and deserves further study.

## Conclusion

The practice of continuous palliative sedation in Portugal is in the range reported in other studies. Delirium was the primary motive for sedation, and midazolam was the most frequently used medication. One point particularly relevant was the low participation of patients and their families in the decision-making process. This process should be more deeply discussed and worked on inside each team. This information may signal alert and reflection in other PC teams concerning their practice.
